# Utilizing Carbon Dots Derived from Waste Face Masks for Pentachlorophenol Detection

**DOI:** 10.1007/s10895-024-03844-0

**Published:** 2024-07-19

**Authors:** Dilek Öztürk, Mahmut Durmuş

**Affiliations:** Department of Chemistry, Gebze Technical University, Gebze, Kocaeli, 41400 Turkey

**Keywords:** Waste facemask, Carbon dot, Pentachlorophenol, Fluorescence sensor

## Abstract

Pentachlorophenol is a very toxic chemical that is used as a pesticide, fungicide, herbicide, wood preservative, etc., and it should be monitored in terms of human health and environmental production. Another environmental problem is the increase in the use of facemasks, especially during the COVID-19 pandemic. This study provides a value added chemicals to sustainability of recycling process. Fluorescent carbon dots (CDs) were synthesized from waste facemasks and investigated their fluorescence sensor performances. UV-Vis and fluorescence spectra of the synthesized carbon dots were recorded in different organic solvents. The sensor properties of these carbon dots against pesticides were investigated, and a ‘turn-off’ response was observed toward pentachlorophenol. The limit of detection was found 8.5 µM in the linear range from 43.3 µM to 375 µM. This study showed that waste plastics such as facemasks can be recycled to obtain carbon dots, which are used in different technological areas such as photocatalysis, bioimaging, etc., as well as in sensors.

**Graphical Abstract:**

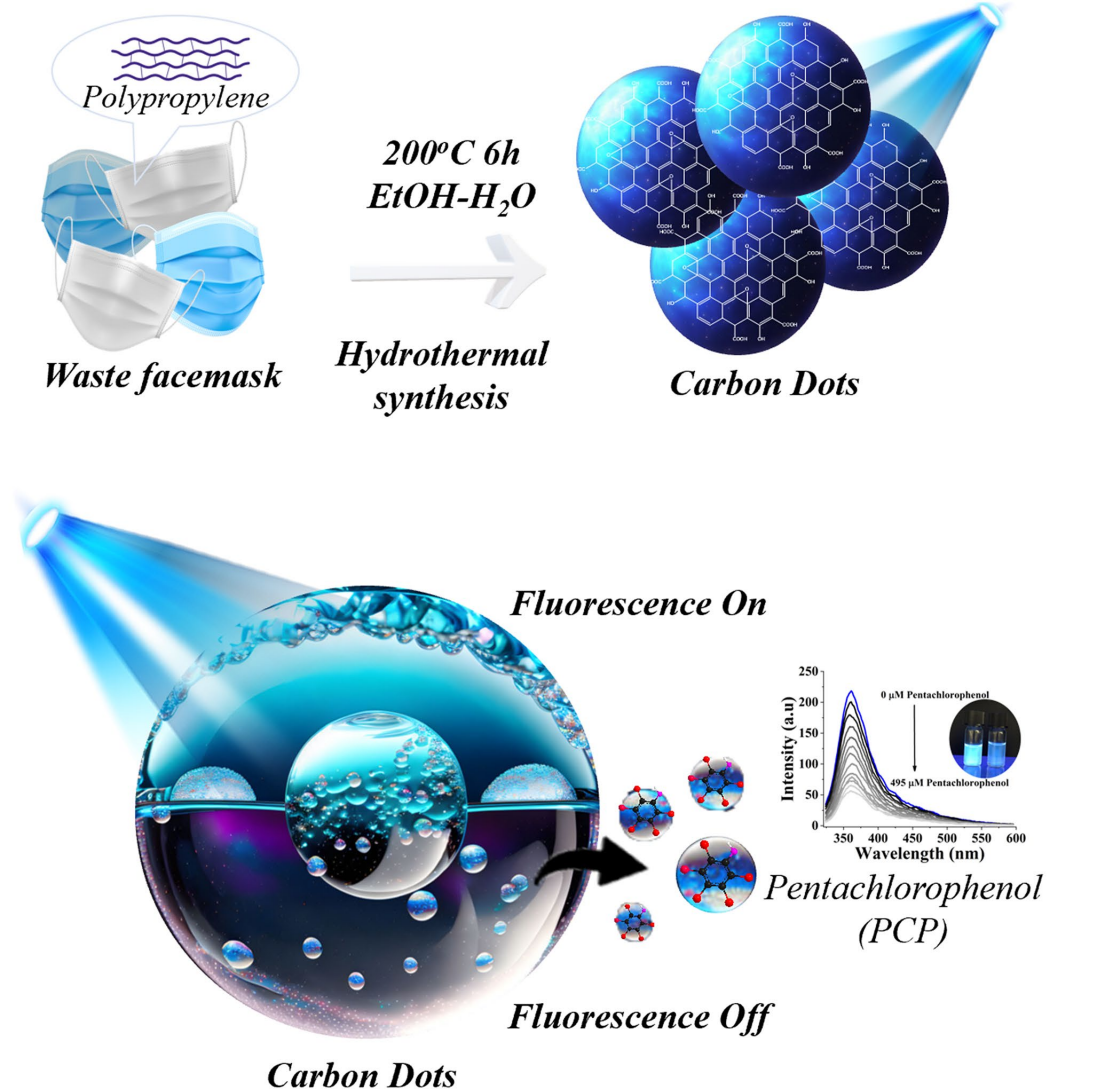

## Introduction

Carbon quantum dots are zero-dimensional fluorescent carbon nanomaterials, and their particle size is less than 10 nm [1]. Due to their outstanding optical properties, excellent photostability, high water solubility, strong chemical resistance, and ease of modification they are used in many technological and advanced applications such as bioimaging, drug release, sensors, optoelectronic devices, and photocatalysis. Nano-sized carbon-based quantum dots are obtained by chemical reactions or by arranging atoms or molecules in a certain order. The production of carbon quantum dots is divided into two different groups according to the starting material: the top-down approach and the bottom-up approach. Laser ablation, ultrasonic treatment, and electrochemical oxidation methods can be examples of top-down methods, and microwave-assisted pyrolysis, thermal decomposition, and hydrothermal approaches can be counted as bottom-up methods [2]. In the top-down process, generally graphene-based materials such as graphene, graphite, carbon nanotubes, carbon nanofibers, etc. are used, and this method is usually about breaking down their structures to get smaller structures. In the bottom-up process, small and aromatic molecules (for example, citric acid, glucose [3], o-phenylenediamine [4], boric acid [5], aromatic hydrocarbons [6] or nanoclusters) are used, and these molecules assemble in a new order. In addition, their size, shape, morphology, and surface conditions can be adjusted [7]. However, they cannot be obtained in the desired amount yet, and their usage is limited due to their high production costs. In order to reduce the production cost and conserve resources, carbon quantum dots are obtained by using different types of waste, such as waste polyethylene terephthalate [8], orange juice [9], wheat straw [10], sugar cane pulp, coffee grounds, eggs and natural renewable biomass sources [11]. The polymer-based wastes are also used in the production of carbon quantum dots [2]. Facemasks are produced from polypropylene [12] and in countries where industrialization has increased, disposable hygienic facemasks have been used for years in order to protect against substances released into the air. Also, facemasks are frequently used in the health sector. Due to the SARS-CoV-2 virus that emerged in Wuhan, China, in 2019, affecting the whole world, the use of facemasks has become mandatory in many countries within the framework of the mask, distance, and cleaning rules. During the COVID-19 pandemic, it has been seen that masks have an active role in protecting people from COVID-19 and other infectious diseases, and disposable facemasks have become even more important in this process. As a result, there is an extraordinary increase in the worldwide production of medical masks. At the same time, these facemasks create a huge waste for the environment, and these wastes need to be recycled or disposed as urgently. Kumari et al. presented a study about conversion of medical plastic waste including disposable syringes, gloves, and face masks to carbon dots in 2024. However, they produced carbon dots in two step process, using thermal calcination at 250^o^C for 3 h followed by the hydrothermal treatment at 180^o^C for 7 h. Their synthesized carbon dots exhibited good biocompatibility and fluorescence turn-off response towards to Fe(ÎII) metal ions [13].In another study, polypropylene surgical masks was used as precursor and acid oxidation process was used for the synthesis of carbon dots. The synthesized carbon dots was investigated for Cr (VI) detection and Cu inhibition mechanism [14]. Similarly, through a top-down approach, carbon dots were synthesized from discarded facemasks using a hydrothermal process, with the addition of 30% v/v hydrogen peroxide [15].

Chlorophenol compounds are highly toxic and carcinogenic chemicals [16]. The increasing number of chlorine atoms on these molecule structures causes the toxic properties of these compounds. Pentachlorophenol (PCP), which is used as a pesticide, fungicide, wood preservative and bactericide, is the most toxic chemical among these chemical compounds [17–19]. Also, this biocide is very stable in the environment and has strong resistance to some treatments, such as photochemical, chemical, and thermal degradation. PCP has low absorption in soil but is transmitted along the food chain, and difficult to degrade this biocide. [20]. For these reasons, PCP is a serious threat to food security and human life. This biocide is identified as a priority pollutant due to its toxicity, according to the US Environmental Protection Agency’s (EPA) report [21]. Therefore, rapid and sensitive analytical methods should be developed to monitor PCP traces. Currently, gas chromatography (GC), gas chromatography-mass spectrometry (GC–MS) [22], high-performance liquid chromatography (HPLC) [23] and electrochemical techniques are used for determination of PCP. However, these techniques are expensive, time-consuming, and complicated. As an alternative to these techniques, fluorescence detection systems have been developed for the determination of PCP due to their low cost, high sensitivity, and ease of operation.

Chen, J. et al. synthesized N, S-doped carbon quantum dots by hydrothermal method from food-derived crawfish shells and showed a fluorescence turn-off-on sensor through pentachlorophenol. Firstly, they quenched the fluorescence of N, S-doped carbon quantum dots using H_2_O_2_ and horseradish peroxidase (HRP), then a turn-on signal was obtained by adding PCP [24]. Wang, H. F. et al. developed an optosensor containing Mn-doped ZnS quantum dots (QDs) on a molecularly imprinted polymer (MIP) layer for PCP detection in water. The detection limit for PCP was found 86 nM, and the recovery of spiked PCP in river water samples ranged from 93 to 106% [25].

In this study, it is aimed to converting waste facemasks into carbon dots by the hydrothermal method, and its optical sensor performances were investigated. Also, this work stands out by obtaining carbon dots in one step without the need for any carbonization process and acid/base treatments. The synthesized carbon dots demonstrated sensitivity to pentachlorophenol (PCP), exhibiting a ‘turn-off’ fluorescence response in the sensor system. Moreover, these carbon dots were capable of directly detecting PCP in environmental samples without the need for any intermediaries. This research offers a straightforward, cost-effective, and eco-friendly approach to repurposing waste face masks, thereby presenting significant potential for advancing ecological recycling practices.

## Materials and Methods

### Material and Equipment

Waste facemasks were collected from the COVID-19 pandemic process in Istanbul, Turkey in between 2021 and 2022 years. Ethanol (99.9%), methanol, DMF, DMSO, and 1,4-dioxane were purchased from Sigma-Aldrich company. Triadimenol, methiocarb, fluopicolide, deltamethrin, triadimefon, iprodione, dimethoate, B-cyfluthrin, fosetyl aluminum, acetamiprid, ethephon, malathion, 2,4-Dichlorophenoxyacetic acid and pentachloro phenol were supplied by Sigma-Aldrich company.

Identification of functional groups was performed by a Perkin Elmer Spectrum FT-IR spectrometer. Absorption spectra were recorded by a Shimadzu 2101 UV-Vis spectrophotometer. Fluorescence emission spectra were obtained by a Varian Eclipse spectrofluorometer. The fluorescence lifetimes were measured using the TCSPC module by Horiba-Jobin-Yvon, Edison, NJ. 3D fluorescence emission spectra were obtained by Horiba-Jobin-Yvon, Edison, NJ. Zeta potential and particle size of synthesized carbon dots were measured by Malvern Zetasizer Nano-ZS and Malvern Mastersizer 2000, respectively. SEM images were recorded by an electron microscope.

### Synthesis of Carbon Dots from Waste Facemasks

Facemasks used during the COVID-19 pandemic process were collected and kept at room temperature for 4 weeks for sterilization. The size of the waste facemask was reduced by using a grinder. Carbon dots were synthesized by modifying the carbon dot synthesis method applied by Aji MP. et al. [2]. Carbon dots from waste facemasks were synthesized using the hydrothermal method. 0.5 gram of waste facemask was placed into a teflon-coated hydrothermal stainless-steel reactor and added 10 mL ethanol-water mixture (1:1 ratio). Then the reaction was carried out at 200^o^C for 6 h. The reaction mixture was passed through a 0.22 μm PTFE filter for remove of large particles from the obtained solution. This solution containing carbon dots was then purified by ultracentrifugation at 13,200 rpm for 30 min. The solution contains the carbon dots was stored at room temperature for sensor measurements. The physical and photophysical characterizations of carbon dots were carried out by particle size, zeta potential measurements, UV-Vis and fluorescence spectroscopic techniques in solution. The carbon dots obtained as powder form by using evaporator at 40^o^C under vacuum from their solution. The morphological characterization of carbon dots was determined by FT-IR, SEM and TEM spectroscopy techniques.

### Fluorescence Quantum Yield of Carbon Dots and Sensor Measurements

The fluorescence quantum yields (Φ_F_) of the synthesized carbon dots were calculated using given Eq. 1 by a comparative method;

1 $${\Phi _F} = {\Phi _F}(Std)\,{{F.\,{A_{Std}}} \over {{F_{Std}}.A}}$$

where F and F_Std_ are the areas under the fluorescence emission curves of CDs and the standard material, respectively. A and A_Std_ are the respective absorbances of CDs and standard material at the excitation wavelengths, respectively. Anthracene was used as a reference material and its fluorescence quantum yields (Φ_Fstd_) is 0.27 in ethanol [26].

After undergoing the necessary purification processes, the carbon dot solution was prepared as a stock solution. From this stock solution, 300 µL was taken and ethanol was added to make the total volume up to 2 mL. This preparation was used to determine the optimal fluorescence intensity and excitation wavelength for sensor measurements. The highest fluorescence intensity was observed at an emission wavelength of 360 nm when the excitation wavelength was set at 310 nm. For the detection process, varying volumes of a 10 mM PCP solution, prepared in ethanol, ranging from 10 µL to 200 µL, were added to the carbon dot solution. After each addition, the fluorescence spectra were recorded. The changes in fluorescence intensity of the carbon dot solution were monitored by sequentially titrating it with the 10 mM PCP solution. Ye Y. et al. used similar detection method for pentachlorophenol using fluorescent metalloporphyrin-based metal-organic frameworks [18].

## Results and Discussion

### Characterization of Carbon Dots

**Fig. 1:**
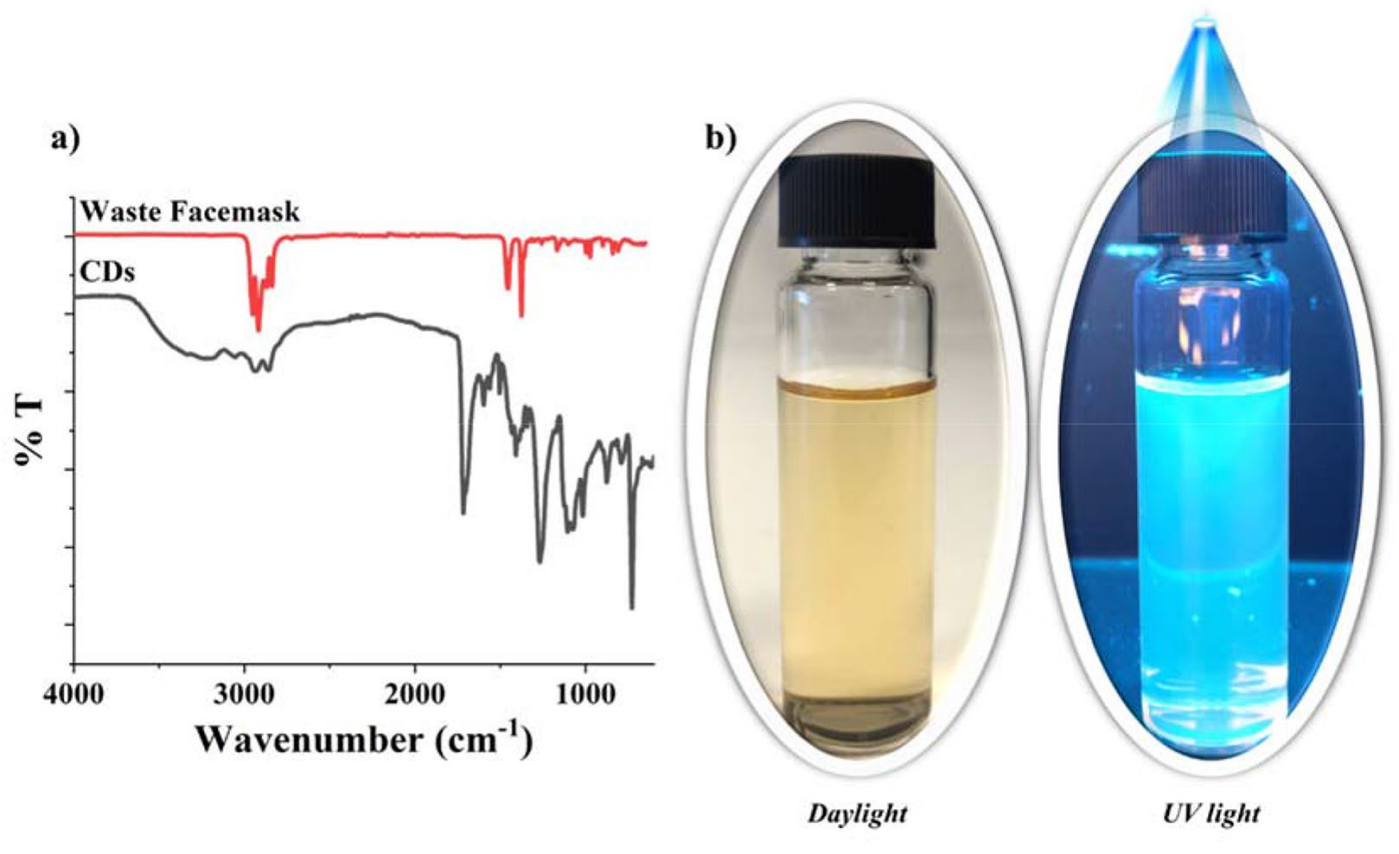
(**a**) FTIR spectra of waste facemask (red line) and synthesized carbon dot from facemask (black line) (**b**) Images of synthesized carbon dot under day light and UV light

The chemical structure of the synthesized carbon dots was determined by FTIR spectroscopy. The FTIR spectra of the waste facemask and carbon dots were given in Fig. 1a. When the peaks in the FTIR spectrum of the waste facemask were compared with the literature, it was revealed that the mask was produced from polypropylene material. The proposed formation mechanism of CDs from polypropylene given in Fig. 5a. Also, the characteristic functional groups of polypropylene, such as -CH_3_ and -CH_2_ were observed at 2935, 2860, 1456 and 1375 cm^− 1^ [1]. According to the FTIR spectrum of the obtained carbon dots, the broad band at around 3300 cm^− 1^ is attributed to –OH groups on the carbon dots. The peak observed at 1682 cm^− 1^ indicated the presence of C = O carbonyl groups in the structure. The peaks at 1117 cm^− 1^ were related to the C-H stretching vibrations. The using heating process breaks the carbon chains in the waste facemask and rearranges them to form carbon dots with a size of < 20 nm [1]. Also, images of synthesized carbon dots under daylight and UV light were shown in Fig. 1b. The carbon dots exhibited blue emission by the light irradiation at 365 nm.

Fluorescence quantum yield is the quantity of fluorescence molecules that is determined as the ratio of emitted photons to absorbed photons. Also, fluorescence quantum yield is desired to have a high value for fluorescence sensor studies.

**Fig. 2:**
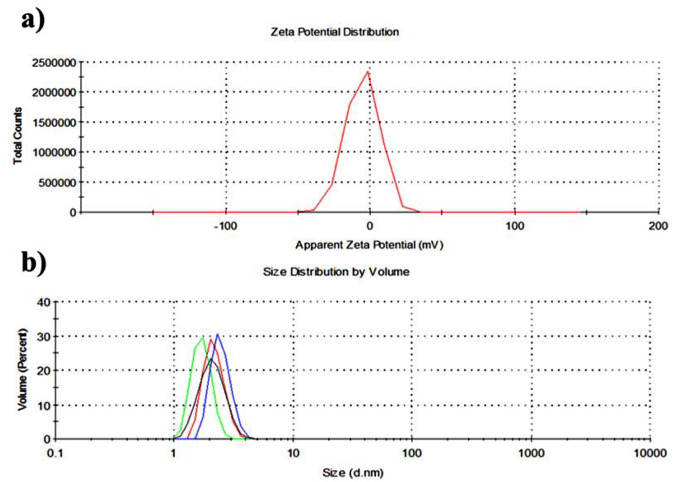
(**a**) Zeta potential and (**b**) particle size analysis of the synthesized carbon dots

The fluorescence quantum yield of synthesized carbon dots was calculated as 0.018 according to the comparative calculation method. Zeta potential analysis gives information about the surface charge of nanoparticles and depends on surface functional groups. A zeta potential outside, the range of -30 mV to + 30 mV is typically regarded as having an adequate level of electrostatic repulsion, which contributes to improved colloidal stability in physical terms [27]. The zeta potential of synthesized carbon dots was measured as -4.77 ± 0.2, as shown in Fig. 2a. That value indicated low anionic functional groups and a tendency to agglomeration [28].

The particle size analysis results of the produced carbon dots were given in Fig. 2b. The synthesized carbon dots showed 1.94 ± 0.28 nm particle size in the ethanol solution.

**Fig. 3:**
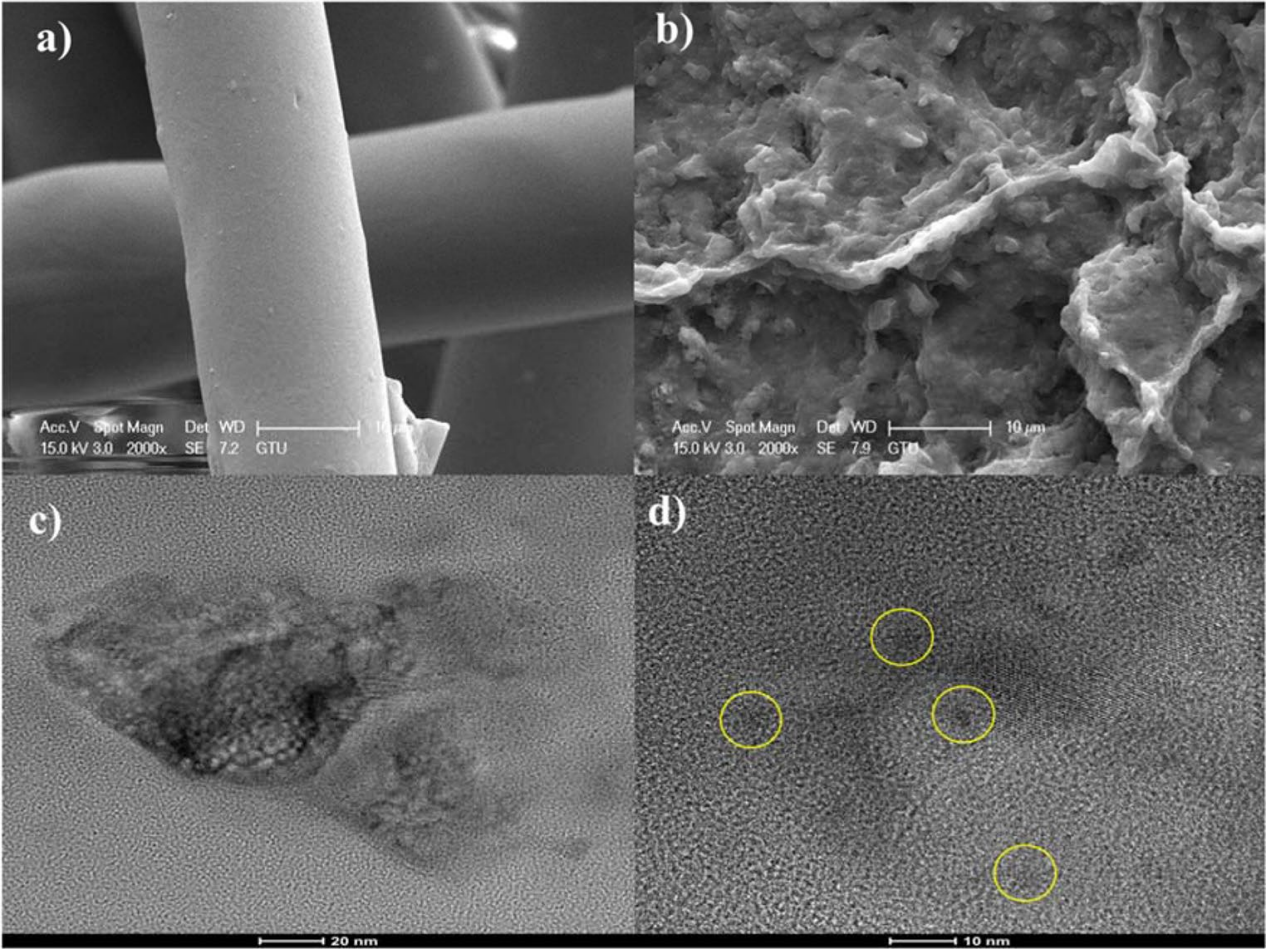
SEM images of (**a**) waste facemask and (**b**) synthesized carbon dots (x10 µm magnification), **c**,**d**) TEM images of synthesized carbon dots

The morphological changes were observed by SEM images of the waste facemask and produced carbon dots in Fig. 3. It can be clearly seen that the structure of the waste facemask was completely disintegrated as a result of the hydrothermal treatment. TEM analysis results of carbon dots also were given in Fig. 3c and Fig. 3d. Particle size analysis in solution form and TEM images were supported each other in particle size distribution.

### Optical Properties of the Produced Carbon Dots

**Fig. 4:**
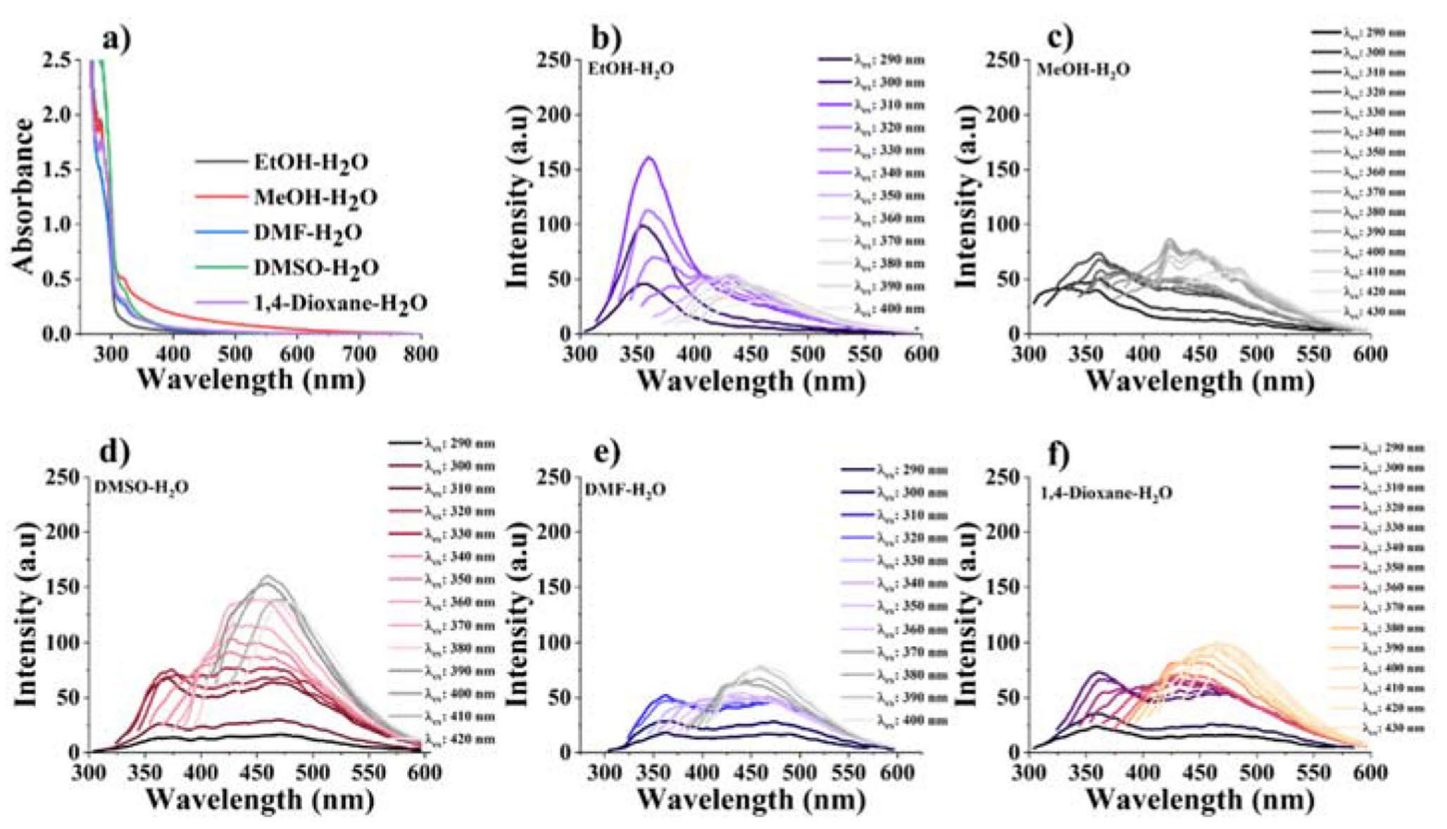
UV-Vis. absorbance and fluorescence emission spectra of the carbon dots in different organic solvent-water mixtures

Quantum dots are known as zero-dimensional structures, and the size of quantum dots is affected by their optical properties [29]. Since their particle size is very small, there is a quantum limitation effect. The unique qualities that emerge from the optical and electronic properties of quantum dots are due to their particle size. The optical properties of the synthesized carbon dots were studied by UV-Vis and fluorescence spectroscopic techniques. The photophysical behavior of carbon dots was examined in different organic solvents such as methanol, DMSO, DMF and 1,4-dioxane using the same solution preparation method. UV-Vis and fluorescence spectra of the synthesized carbon dots in different organic solvent-water mixtures were given in Fig. 4. UV-Vis spectra were recorded in different organic solvent-water mixtures such as ethanol, methanol, DMF, DMSO, and 1,4-dioxane. Two absorption peaks were observed at 284 and 320 nm in all solvents. The strong absorption peak at 284 nm resulted from π – π* transition of the sp^2^ C = C aromatic bond in carbon dot structure. The synthesized carbon dots exhibited different fluorescence emission behaviours in different organic solvents. Carbon dots often display excitation wavelength-dependent behavior in organic solvents. This variability arises from their diverse sizes and the multiple functional groups on the structure (carboxyl, hydroxyl and carbonyl groups), which can create different emissive sites [30]. Fluorescence emission spectra were obtained by changing the excitation wavelengths from 290 nm to 400 nm in all solvents. The synthesized carbon dots exhibited sharper, smoother, and higher fluorescence intensity in ethanol, whereas they exhibited noisy, and low fluorescence intensity in other solvents. The fluorescence emission of carbon dots showed the highest intensity when excited at 360 nm in a polar protic solvent such as ethanol. However, in polar aprotic solvents such as DMSO, DMF, and 1,4-dioxane, the fluorescence emission shifted to 450 nm. These photophysical results indicated that the interactions between the carbon dots and the solvents lead to bathochromic (red shift) and hypsochromic (blue shift) effects, which played a crucial role in determining the emission wavelength [31]. An ethanol-water mixture was chosen to obtain more meaningful results in sensor measurements.

### Sensor Performance of the Synthesized Carbon Dots

**Fig. 5:**
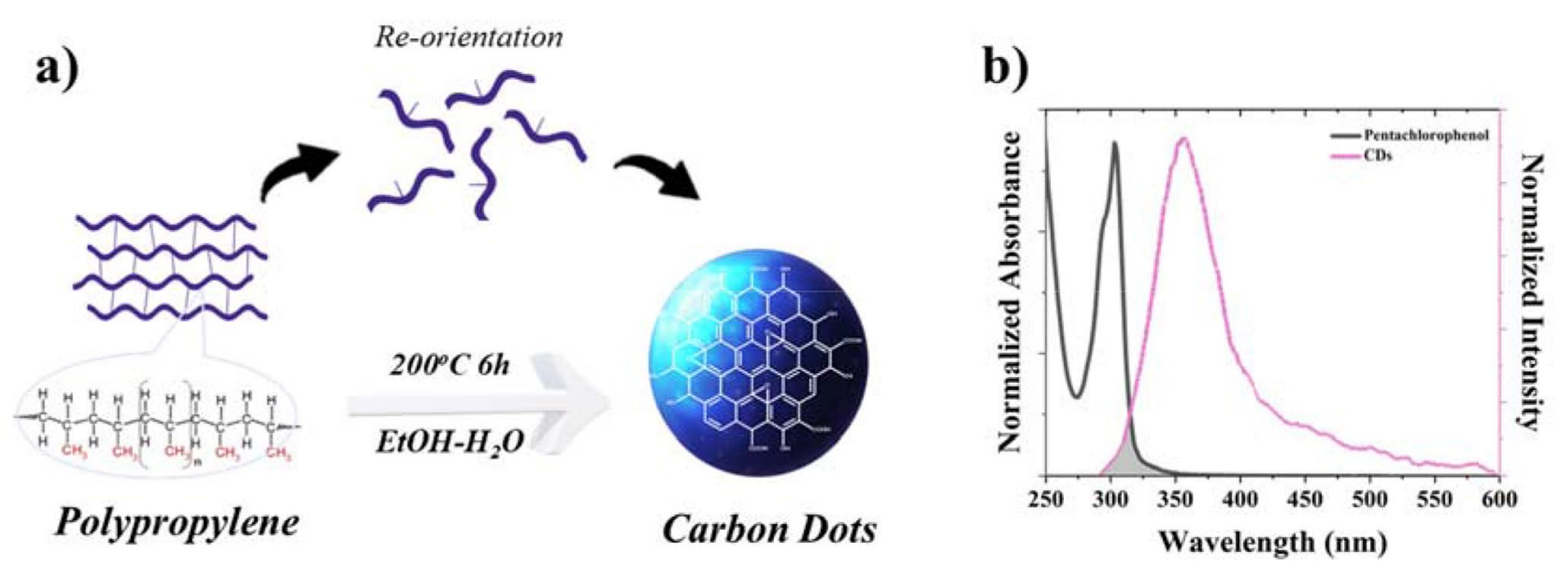
(**a**) Proposed formation mechanism of carbon dots (CDs) from waste face mask materials (**b**) Overlay graph for the emission spectrum of CDs and the absorption spectrum of pentachlorophenol

**Fig. 6:**
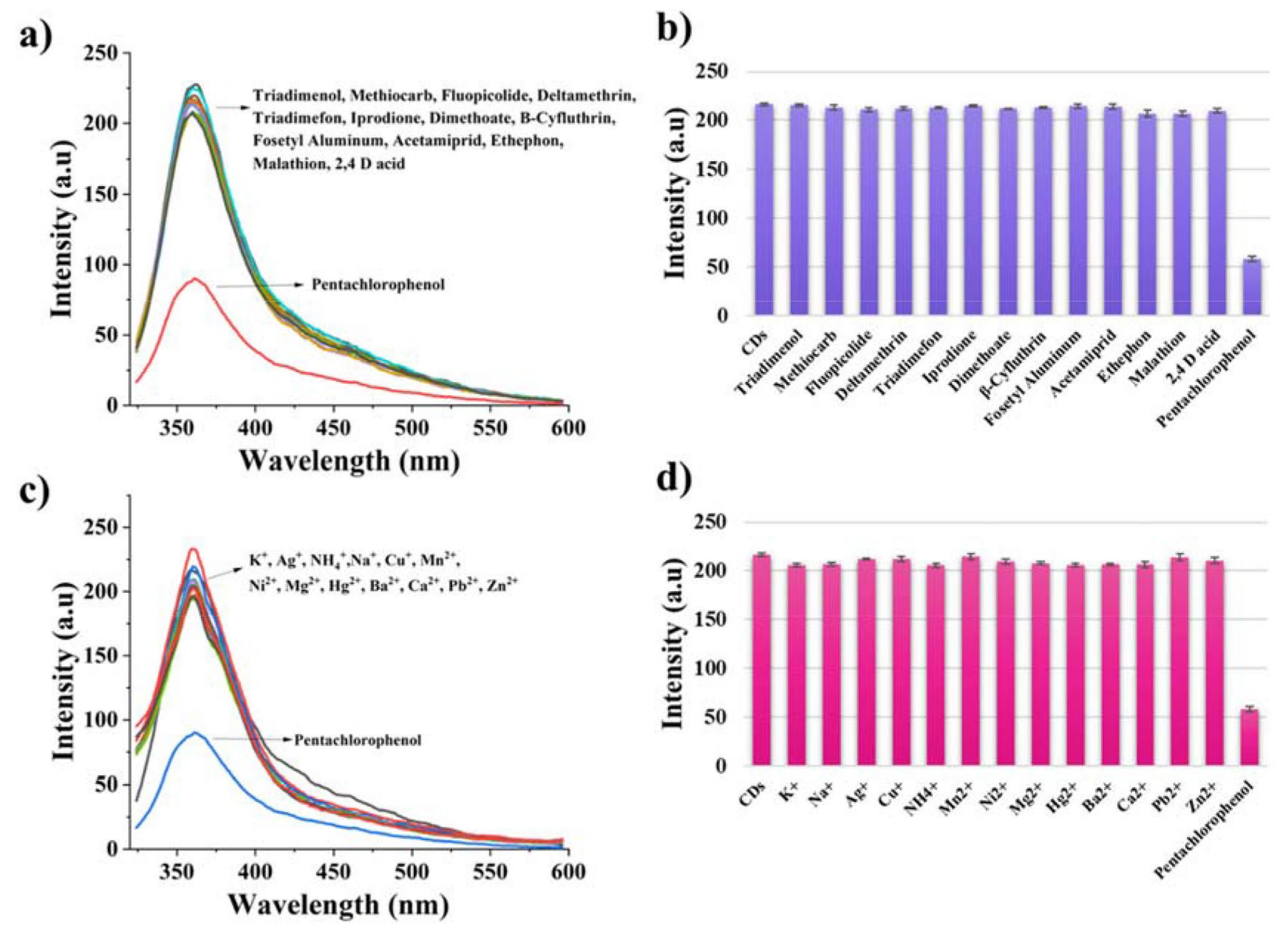
Selectivity behaviour of the synthesized carbon dots against different (**a**), (**b**) pesticides and (**c**), (**d**) metal ions (All selectivity analytes were used in equivalent 0.10 M water/ethanol solutions.)

The fluorescence sensor performance of the synthesized carbon dots was investigated using pesticides as analytes. These carbon dots were found sensitive to pentachlorophenol among used thirteen different pesticides, including triadimenol, methiocarb, fluopicolide, deltamethrin, triadimefon, iprodione, dimethoate, B-cyfluthrin, fosetyl aluminium, acetamiprid, ethephon, malathion, and 2,4 D-acid and several metal ions. When comparing the fluorescence intensity spectra of pentachlorophenol and other pesticides, it can be seen that the quenching efficiency of pentachlorophenol could be more effective than other pesticides used in this study. This quenching mechanism may be explained by the Förster Resonance Energy Transfer (FRET) mechanism between PCP and CDs. Quenching mechanism of carbon dots especially in biosensing studies could be dynamic quenching, static quenching, photoinduced electron transfer (PET), energy transfer, and inner filter effect (IFE) [32]. FRET is an electrodynamic phenomenon describable by classical physics principles. It takes place between CDs in an excited state and a ground-state quencher, provided that the emission spectrum of the CDs overlaps with the absorption spectrum of the quencher. This energy transfer occurs without the emission of a photon, facilitated by long-range dipole-dipole interactions between the CDs and the quencher [33]. PCP has three absorption bands at 220, 251 and 321 nm [34] and CDs have an emission band 360 nm in ethanol-water solvent. The spectral overlapping between the emission of CDs and the absorption of PCP molecules explained the FRET mechanism [35]. The FRET mechanism between PCP and CDs was shown in Fig. 5b. A partial overlap was observed between the absorption peak of PCP at 304 nm and the emission spectrum of carbon dots at 360 nm when PCP was acted upon as the acceptor and carbon dots were utilized as the donor fluorophore. The selectivity behaviour of the synthesized carbon dots through the use of pesticides were given in Fig. 6.

**Fig. 7:**
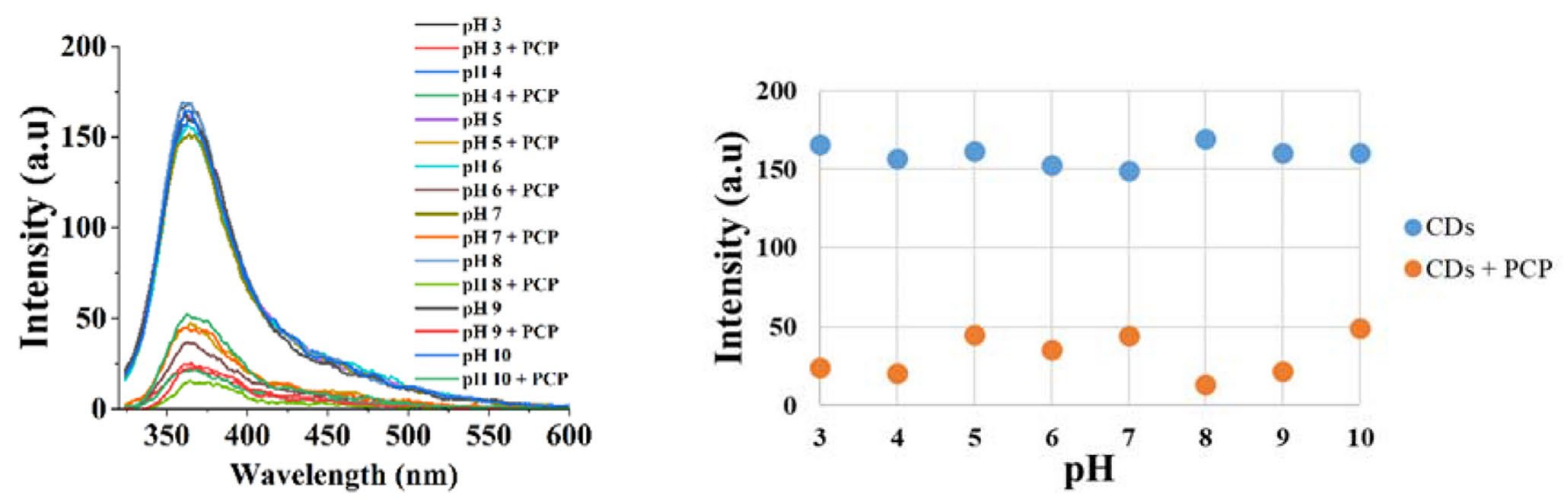
pH effect on fluorescence sensor performance of carbon dots for pentachlorophenol detection

The pH of a medium can change due to various factors, such as biological processes or chemical reactions. This parameter can have a significant impact on the performance of fluorescence sensors. Also, pH drifts can impact the accuracy and reliability of fluorescence sensor measurements. In this study, the pH of the medium was changed from 3 to 10 using a Britton-Robinson buffer solution and examined influence the fluorescence signal of PCP. According to Fig. 7, the fluorescence signal of CDs showed low changes in all pH media. The fluorescence signal is less affected in the presence of PCP at pH 5, 6, and 10 than others. pH 8 exhibited the highest quenching medium.

**Fig. 8:**
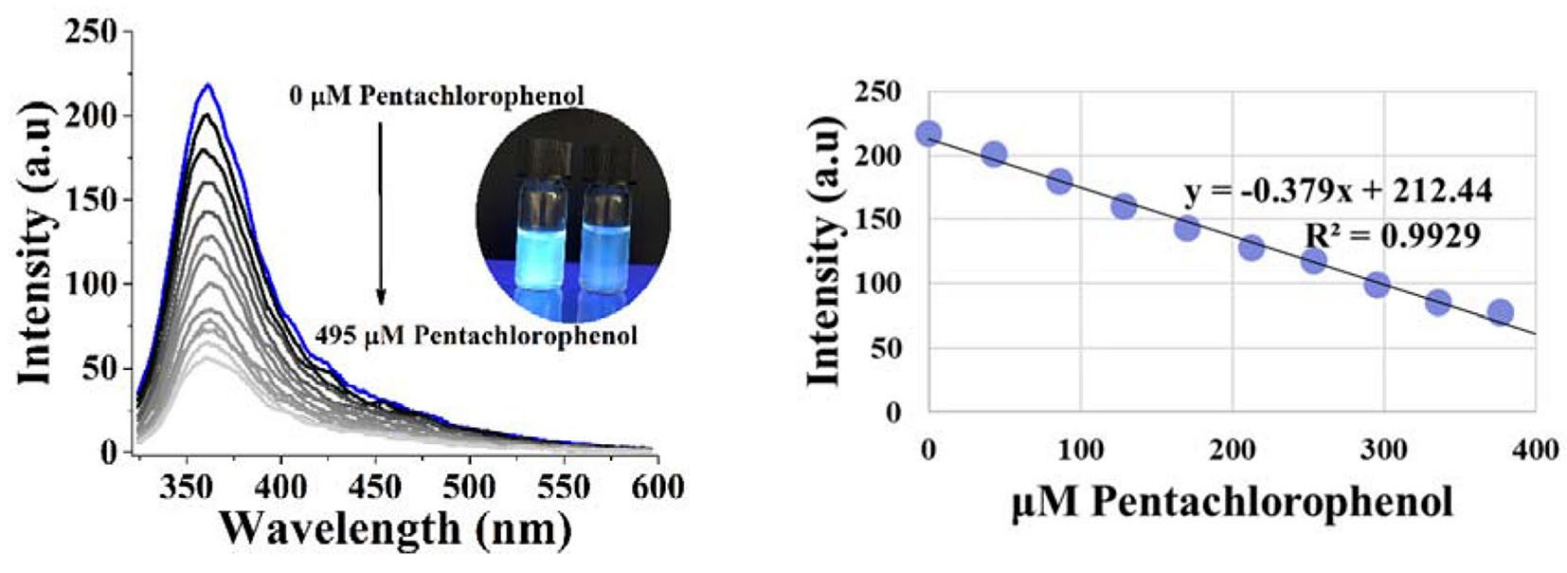
Fluorescence intensity changes against different concentrations of pentachlorophenol

Fluorescence intensities of carbon dots at different concentrations of pentachlorophenol were measured to determine the sensitivity. As shown in Fig. 8, fluorescence emission intensity decreased upon addition of pentachlorophenol solution, by the concentration increasing from 43.3 µM to 375 µM. A linear relationship was observed between fluorescence intensity and concentration of pentachlorophenol (R^2^ = 0.9929). The linear equation was found as y=-0.379x + 212.44, the limit of detection value was calculated as 8.5 µM based on three times the standard deviation rule (LOD = 3Std/S), and the limit of quantification value was calculated as 25.7 µM based on ten times the standard deviation rule (LOQ = 10Std/S).

**Fig. 9:**
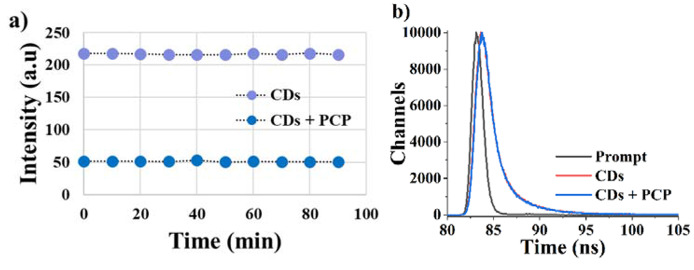
(**a**) Photostability of CDs and CDs + PCP, (**b**) Fluorescence lifetimes of CDs and CDs + PCP (laser excitation source: 390 nm nanoled)

Photostability plays a crucial role in sensor systems. This parameter must be checked for the accuracy of the results in the developed fluorescence sensor systems. For this purpose, a photostability study was performed for CDs and CDs-PCP systems between 0 and 90 min under daylight (Fig. 9a). The fluorescence intensity of CDs and CDs-PCP was unaffected by light and stayed at nearly the same values until 90 min. Consequently, CDs and CDs-PCP systems have high photostability.

Fluorescence quenching is a phenomenon in which the emission intensity of a fluorophore is reduced or “quenched” due to the presence of a quencher molecule. This mechanism occurs in two types: static or dynamic quenching. Dynamic quenching involves the collisional interaction between the excited fluorophore and the quencher molecule. The excited-state fluorophore transfers energy to the quencher through non-radiative processes, such as electron transfer or energy transfer. Static quenching involves the formation of a non-fluorescent ground-state complex between the fluorophore and the quencher. The excited state lifetime of the fluorophore is reduced in the collisional quenching mechanism [36].

Fluorescence lifetime measurements of CDs and CDs + PCP were given in Fig. 9b. CDs have two different lifetimes of 1 (76%) and 4 (24%) ns, which indicates two different fluorophores found in the CDs fluorescence system. When CDs were quenched with PCP, the lifetimes of CDs + PCP systems did not show any changes. The observed outcomes support the notion that the quenching system operates through a static mechanism.

**Fig. 10:**
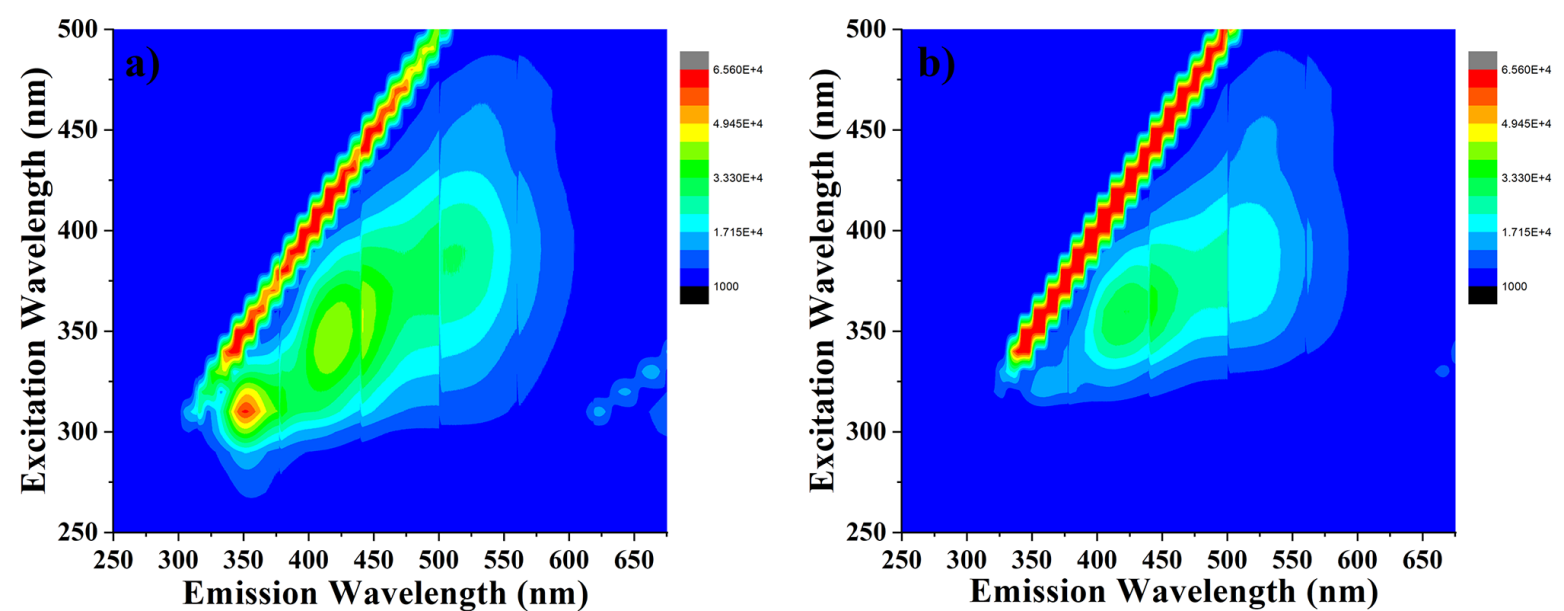
3D fluorescence counter plots of (**a**) CDs and (**b**) CDs + PCP

3D fluorescence spectra are useful for characterization of the surroundings of fluorescence molecules and better understanding the behaviour of molecules [37]. 3D fluorescence counter plots of CDs and CDs-PCP were given in Fig. 10. As can be seen from these figures, CDs have a broad emission band, and only the strong emission band at 360 nm disappears in the presence of PCP.

The synthesized carbon dots from waste facemasks were highly sensitive to pentachlorophenol and can be applied to the detection of pentachlorophenol on real samples. The effectivity of the developed fluorescence sensor was evaluated in our laboratory tap water. The spike and recovery analyses were performed at different concentrations without any pre-treatment process. The recovery of the spiked tap water sample was found as 106.9%, and this result can be employed as a promising sensor for pentachlorophenol in real water samples. The sensors developed using quantum dots in the literature and their analytical performance parameters for PCP detection are given in Table 1. The table shows that there are some studies obtained lower detection limits. The main gain of this study, when compared to the earlier studies, is that the fluorescence-based sensor system was obtained from waste facemasks and the obtained carbon dots were used for the determination of PCP, which is a highly toxic chemical.

**Table 1:** Analytical parameters of the developed carbon dots sensors for PCP detection

Materials	Detection method	Sample	LOD	Linear range	Added	Found	% Recovery	Ref.
CDs	Fluorescence	Tap water	8.5 µM	43.3–375 µM	417.7 µM	446.8 ± 1.3 µM	106.9	This work
N, S-doped CQDs	Fluorescence	Lake water	2.30 µM	10–300 µM	100 µM	105.17 µM	105.2	[24]
CQDs	Electrogenerated chemiluminescence	Tap water	1.3 × 10^− 12^ g L^− 1^	10 pg L^− 1^ -1.0 µg L^− 1^	100 ng L^− 1^	103.59 ng L^− 1^	103.59	[38]
ZnSe QDs decorated MWCNT	Electrochemical	Fish meat	2.0 × 10^− 9^ mol L^− 1^	8.0 × 10^− 8^ to 4.0× 10^− 6^ mol L^− 1^	2.0 × 10^− 7^ mol L^− 1^	1.79 × 10^− 7^ mol L^− 1^	89.5	[39]
N-doped GQDs	Electrochemiluminescence	Tap water	2 × 10^− 17^ g mL^− 1^	1 × 10^− 15^ to 1 × 10^− 5^ g mL^− 1^	5 pg mL^− 1^	5.13 ± 0.15 pg mL-1	102.6	[40]

## Conclusion

Waste facemasks have been successfully transformed into carbon dots as a new generation material by the hydrothermal method for the first time in this study. Carbon dots were obtained as a particle size of 1.94 ± 0.28 nm and maximum emission at 360 nm in ethanol. Its physical and chemical structure was characterized by UV-Vis, fluorescence, FTIR spectra, and the morphological characterization was determined by particle size, SEM and TEM analysis. The fluorescence sensor performance of the synthesized carbon dots was investigated using pesticides as analytes. Analytical parameters were performed by giving a ‘’turn-off’’ fluorescence sensor response in the presence of pentachlorophenol. The detection limit was found to be 8.5 µM in the linear range of 43.3–375 µM. Also, spike and recovery analyses were investigated on tap water as a real sample. This study is a promising work indicating that waste polymer materials can be recycled and reused. According to this research, using waste materials with similar properties contributes to new application areas. Additionally, it increases the number of optical studies by contributing to the limited number of fluorescence-based sensor studies for PCP detection in the literature. In this study, carbon dots were synthesized from waste polymer polypropylene material in a single step, and PCP detection was determined by a turn-off signal.

## Data Availability

No datasets were generated or analysed during the current study.

## References

[1] Ding H, Zhou X-X, Wei J-S, Li X-B, Qin B-T, Chen X-B, Xiong H-M (2020) Carbon dots with red/near-infrared emissions and their intrinsic merits for biomedical applications. Carbon 167:322–344

[2] Aji MP, Wati AL, Priyanto A, Karunawan J, Nuryadin BW, Wibowo E, Marwoto P, Sulhadi S (2018) Polymer carbon dots from plastics waste upcycling. Environ Nanotechnol Monit Manag 9:136–140

[3] Hallaj T, Amjadi M, Manzoori JL, Shokri R (2015) Chemiluminescence reaction of glucose-derived graphene quantum dots with hypochlorite, and its application to the determination of free chlorine. Microchim Acta 182:789–796

[4] Wang J, Zhu Y, Wang L (2019) Synthesis and applications of red-emissive carbon dots. Chem Rec 19:2083–2094 10.1002/tcr.201800172 30762933

[5] Wang L, Li W, Yin L, Liu Y, Guo H, Lai J, Han Y, Li G, Li M, Zhang J, Vajtai R, Ajayan PM, Wu M (2020) Full-color fluorescent carbon quantum dots. Sci Adv 6:eabb6772 33008913 10.1126/sciadv.abb6772 PMC7852397

[6] Zhou L, Geng J, Liu B (2013) Graphene quantum dots from polycyclic aromatic hydrocarbon for bioimaging and sensing of Fe3+ and hydrogen peroxide. Part Part Syst Charact 30:1086–1092

[7] Facure MHM, Schneider R, Lima JBS, Mercante LA, Correa DS (2021) Graphene quantum dots-based nanocomposites applied in electrochemical sensors: a recent survey. Electrochem 2:490–519

[8] Hu Y, Gao Z, Yang J, Chen H, Han L (2019) Environmentally benign conversion of waste polyethylene terephthalate to fluorescent carbon dots for on-off-on sensing of ferric and pyrophosphate ions. J Colloid Interface Sci 538:481–488 30537661 10.1016/j.jcis.2018.12.016

[9] Sahu S, Behera B, Maiti TK, Mohapatra S (2012) Simple one-step synthesis of highly luminescent carbon dots from orange juice: Application as excellent bio-imaging agents. Chem Commun 48:8835–8837 10.1039/c2cc33796g 22836910

[10] Yuan M, Zhong R, Gao H, Li W, Yun X, Liu J, Zhao X, Zhao G, Zhang F (2015) One-step, green, and economic synthesis of water-soluble photoluminescent carbon dots by hydrothermal treatment of wheat straw, and their bio-applications in labeling, imaging, and sensing. Appl Surf Sci 355:1136–1144

[11] Hu G, Ge L, Li Y, Mukhtar M, Shen B, Yang D, Li J (2020) Carbon dots derived from flax straw for highly sensitive and selective detections of cobalt, chromium, and ascorbic acid. J Colloid Interface Sci 579:96–108 32574732 10.1016/j.jcis.2020.06.034

[12] Aragaw TA (2020) Surgical face masks as a potential source for microplastic pollution in the COVID-19 scenario. Mar Pollut Bull 159:111517 32763564 10.1016/j.marpolbul.2020.111517 PMC7381927

[13] Kumari M, Chaudhary GR, Chaudhary S (2024) Transformation of medical plastic waste to valuable carbon dots: a sustainable recycling of medical waste to efficient fluorescent marker. J Mol Liq 395:123910

[14] Miao C, Wang Q, Yang S, Tang Y, Liu X, Lu S (2024) Hydrothermal route upcycling surgical masks into dual-emitting carbon dots as ratiometric fluorescent probe for cr (VI) and corrosion inhibitor in saline solution. Talanta 275:126070 38678920 10.1016/j.talanta.2024.126070

[15] Arrigo A, Cancelliere AM, Galletta M, Burtone A, Lanteri G, Nastasi F, Puntoriero F (2023) From waste to energy: luminescent solar concentrators based on carbon dots derived from surgical facemasks. Mater Adv 4:5200–5205

[16] Ling W (2020) A highly sensitive and selective electrochemical sensor for pentachlorophenol based on reduced graphite oxide-silver nanocomposites. Food Anal Methods 13:2050–2058

[17] Ren Y, Ma J, Lee Y, Han Z, Cui M, Wang B, Long M, Khim J (2021) Reaction of activated carbon zerovalent iron with pentachlorophenol under anaerobic conditions. J Clean Prod 297:126748

[18] Ye Y, Huang C, Yang J, Li Y, Zhuang Q, Gu J (2019) Highly selective and rapid detection of pentachlorophenol in aqueous solution with metalloporphyrinic MOFs. Microporous Mesoporous Mater 284:36–42

[19] Meskher H, Achi F, Moussa FB, Henni A, Belkhelfa H (2023) A novel pentachlorophenol electrochemical sensor based on nickel-cobalt layered double hydroxide doped with reduced graphene oxide composite. ECS Adv 2:016503

[20] Basova T, Hassan A, Yuksel F, Gürek AG, Ahsen V (2010) Optical detection of pentachlorophenol in water using thin films of octa-tosylamido substituted zinc phthalocyanine. Sens Actuators B 150:523–528

[21] Khuzwayo Z, Chirwa EMN (2017) The impact of alkali metal halide electron donor complexes in the photocatalytic degradation of pentachlorophenol. J Hazard Mater 321:424–431 27669383 10.1016/j.jhazmat.2016.08.069

[22] Valdez CA, Salazar EP, Leif RN (2022) Trimethyloxonium-mediated methylation strategies for the rapid and simultaneous analysis of chlorinated phenols in various soils by electron impact gas chromatography–mass spectrometry. Sci Rep 12:1401 35082365 10.1038/s41598-022-05463-w PMC8792036

[23] Fan C, Li N, Cao X (2015) Determination of chlorophenols in red wine using ionic liquid countercurrent chromatography as a new pretreatment method followed by high-performance liquid chromatography. J Sep Sci 38:2109–2116 25826668 10.1002/jssc.201500172

[24] Chen J, Xia X, Li P, Yu H, Xie Y, Guo Y, Yao W, Qian H, Cheng Y (2023) A facile off–on fluorescence sensor for pentachlorophenol detection based on natural N and S co-doped carbon dots from crawfish shells. Food Chem 405:134802 36371832 10.1016/j.foodchem.2022.134802

[25] Wang H-F, He Y, Ji T-R, Yan X-P (2009) Surface molecular imprinting on Mn-doped ZnS quantum dots for room-temperature phosphorescence optosensing of pentachlorophenol in water. Anal Chem 81:1615–1621 19170523 10.1021/ac802375a

[26] Dawson WR, Windsor MW (1968) Fluorescence yields of aromatic compounds. J Phys Chem 72:3251–3260

[27] Joseph E, Singhvi G (2019) In: Grumezescu AM (ed) In nanomaterials for drug delivery and therapy. William Andrew Publishing, pp 91–116

[28] Samimi S, Maghsoudnia N, Eftekhari RB, Dorkoosh F (2019) In: Mohapatra SS, Ranjan S, Dasgupta N, Mishra RK, Thomas S (eds) Characterization and biology of nanomaterials for drug delivery. Elsevier, p 47–76

[29] Jamieson T, Bakhshi R, Petrova D, Pocock R, Imani M, Seifalian AM (2007) Biological applications of quantum dots. Biomater 28:4717–4732 17686516 10.1016/j.biomaterials.2007.07.014

[30] Zhu S, Song Y, Zhao X, Shao J, Zhang J, Yang B (2015) The photoluminescence mechanism in carbon dots (graphene quantum dots, carbon nanodots, and polymer dots): current state and future perspective. Nano Res 8:355–381

[31] Mohammad-Jafarieh P, Akbarzadeh A, Salamat-Ahangari R, Pourhassan-Moghaddam M, Jamshidi-Ghaleh K (2021) Solvent effect on the absorption and emission spectra of carbon dots: evaluation of ground and excited state dipole moment. BMC Chem 15:53 34563252 10.1186/s13065-021-00779-6 PMC8587513

[32] Iqbal A, Tian Y, Wang X, Gong D, Guo Y, Iqbal K, Wang Z, Liu W, Qin W (2016) Carbon dots prepared by solid state method via citric acid and 1,10-phenanthroline for selective and sensing detection of Fe2 + and Fe3+. Sens Actuators B 237:408–415

[33] Zu F, Yan F, Bai Z, Xu J, Wang Y, Huang Y, Zhou X (2017) The quenching of the fluorescence of carbon dots: a review on mechanisms and applications. Microchim Acta 184:1899–1914

[34] Garbellini GS, Salazar-Banda GR, Avaca LAJPEA (2010) Effects of ultrasound on the degradation of pentachlorophenol by boron-doped diamond electrodes. Port Electrochim Acta 28:405–415

[35] Tang C, Meng G, Huang Q, Huang Z, Zhang X, Wang M (2012) A silica xerogel thin film based fluorescent sensor for pentachlorophenol rapid trace detection. Sens Actuators B 171–172:332–337

[36] Lichota A, Szabelski M, Krokosz A (2022) Quenching of protein fluorescence by Fullerenol C60(OH)36 nanoparticles. Int J Mol Sci 23:12382 10.3390/ijms232012382 PMC9603995 36293241

[37] Tümay SO, Şenocak A, Çoşut B, Alidağı HA, Yeşilot S (2023) A water-soluble small molecular fluorescent sensor based on phosphazene platform for selective detection of nitroaromatic compounds. Photochem Photobiol Sci 22:1429–1444 36807055 10.1007/s43630-023-00388-3

[38] Li J, Wang N, Tran TT, Huang Ca, Chen L, Yuan L, Zhou L, Shen R, Cai Q (2013) Electrogenerated chemiluminescence detection of trace level pentachlorophenol using carbon quantum dots. Analyst 138:2038–2043 23391969 10.1039/c3an36653g

[39] Feng S, Yang R, Ding X, Li J, Guo C, Qu L (2015) Sensitive electrochemical sensor for the determination of pentachlorophenol in fish meat based on ZnSe quantum dots decorated multiwall carbon nanotubes nanocomposite. Ionics 21:3257–3266

[40] Luo L, Li L, Xu X, Liu D, Li J, Wang K, You TJRA (2017) Determination of pentachlorophenol by anodic electrochemiluminescence of Ru(bpy)32 + based on nitrogen-doped graphene quantum dots as co-reactant. RSC Adv 7:50634–50642

